# The Issue of Proxies and Choice Architectures. Why EU Law Matters for Recommender Systems

**DOI:** 10.3389/frai.2022.789076

**Published:** 2022-04-28

**Authors:** Mireille Hildebrandt

**Affiliations:** ^1^Institute of Computing and Information Sciences (iCIS), Science Faculty, Radboud University, Nijmegen, Netherlands; ^2^Research Group Law Science Technology & Society (LSTS), Faculty of Law and Criminology, Vrije Universiteit Brussel, Brussels, Belgium

**Keywords:** micro-targeting, machine learning, behavioral profiling, political economy, behaviorism, Goodhart effect, affordance, Brussels effect

## Abstract

Recommendations are meant to increase sales or ad revenue, as these are the first priority of those who pay for them. As recommender systems match their recommendations with inferred preferences, we should not be surprised if the algorithm optimizes for lucrative preferences and thus co-produces the preferences they mine. This relates to the well-known problems of feedback loops, filter bubbles, and echo chambers. In this article, I discuss the implications of the fact that computing systems necessarily work with proxies when inferring recommendations and raise a number of questions about whether recommender systems actually do what they are claimed to do, while also analysing the often-perverse economic incentive structures that have a major impact on relevant design decisions. Finally, I will explain how the choice architectures for data controllers and providers of AI systems as foreseen in the EU's General Data Protection Regulation (GDPR), the proposed EU Digital Services Act (DSA) and the proposed EU AI Act will help to break through various vicious circles, by constraining how people may be targeted (GDPR, DSA) and by requiring documented evidence of the robustness, resilience, reliability, and the responsible design and deployment of high-risk recommender systems (AI Act).

## Introduction

Recommender systems (RecSyss) based on collaborative filtering inevitably create feedback loops, echo chambers, and filter bubbles, because algorithms cannot be trained on future data. On top of that, the political economy that drives the incentive structure for providers and users of these systems creates perverse incentives, skewing the inferences in a direction that is favorable for those hoping to make a profit or win or confuse public opinion. Both issues are exacerbated by the fact that computing systems necessarily work with proxies when determining the target outcome (relevant recommendations) and when deciding on relevant feature variables (based on their distribution in the training data).

This article puts forward two reasons why the issue of proxies is difficult to resolve. First, to resolve means first to accept that any inferred recommendation will always be limited by the choice of the proxies; the issue cannot be resolved by selecting perfect proxies that are identical with what they aim to map. Second, the issue can be mitigated by selecting better proxies, but only if “better” relates to better attention paid to perverse economic incentives and to more acuity as to the disturbingly naïve behaviorist assumptions that plague the research design of collaborative filtering based on behavioral data. The first requires more research into the political economy that informs the research design of RecSyss, the second requires more research into the way the output of RecSyss is presented, notably the use of performance metrics that glean over the nature of computable proxies and their limitations. This article thus makes two points: (1) one at the level of epistemology, regarding the assumptions that inform objectivist accounts of what RecSyss actually do and (2) one at the level of the political economy, demonstrating how current economic incentives drive design decisions in the domain of RecSyss.

The current and upcoming EU legislative framework creates new choice architectures for users (deployers) and providers of RecSyss, such that end-users' agency is taken seriously, and their capabilities are enhanced rather than captured in loops, bubbles and echo chambers. At the end of the day, these new choice architectures will not only constrain how RecSyss can be designed and developed; they will instigate a kind of “by design” protection that is based on carefully selected checks and balances, meant to create a computational infrastructure that is not poised toward manipulation but toward informed interaction. This will require upfront involvement of the developers of these systems, who need to understand that law is not a boring bag of rules meant to constrain them but a set of dedicated incentives to build more resilient, robust, reliable, and responsible RecSyss. In the final part of the article it should become clear how both the GDPR and the AI Act impose constraints capable of relieving developers from the perverse incentives that force them to not only cater to “mined” preferences but to also influence preferences in order to increase so-called “user engagement” (Zou et al., 2019). Qualifying these incentives as “perverse” highlights the fact that the outcome of such incentives flies in the face of what one would normally expect from a trustworthy recommender system^1^; instead of attuning recommendations to assumedly given user preferences these systems attune their recommendations so as to change user preferences into what is profitable for the platform that benefits from such changes. In itself this observation is not new, but the relationship between, on the one hand, the political economy that drives the design of these systems when deployed in the real world and, on the other hand, design decisions regarding proxies, has not been made in this way.

In this article, I focus on RecSyss that combine the objective of providing recommendations in whatever context with a business model that aims to persuade the end-user to prioritize and/or develop specific preferences, for instance resulting in said “user engagement” or in selection of products from advertisers that pay a higher conversion fee (Viljoen et al., 2021). This is not to suggest that systems not geared toward such persuasion are neutral or objective—relevance always depends on purpose, context and agent; in that sense even a well-designed and properly deployed system is biased by definition, and this is not a bug but a feature (Hildebrandt, 2021a).

Those eager to check out the potential impact of the EU legislative framework can start with the sections “the intermezzo on counter profiling” (for the relevance of the Digital services act or DSA)^2^ and “the legal framework as a choice architecture” (for the relevance of the General data protection regulation or GDPR^3^ and the proposed AI Act)^4^. They should, however, take into account that the article is not merely a legal analysis but aims to situate how and why law matters here. This means the legal analysis is focused on how the GDPR and the proposed DSA and AI Act re-configure the choice architecture of big tech in relation to the risks that RecSyss pose for human rights. The key point of the article is, on the one hand, to better understand the issues of proxies that infest RecSyss and, on the other hand, to better understand the nature of law as a means to protect individual human beings against some of the detrimental effects of the pseudo-science that underpins many of the currently marketed RecSyss, notably where such effects are skewed against those already disadvantaged.

In “defining RecSyss,” I will put my cards on the table as to how I understand the role and function of RecSyss, emphasizing that they constitute an inevitable filtering mechanism since we moved from information scarcity to information overflow. As Dewey explained, instruments are not neutral (Dewey, 1988). They can be developed and used in different ways that will reconfigure the goals they aim to achieve, even as the goals co-determine the instruments to achieve them. This also goes for RecSyss. In “a political economy of recommender systems,” I will explain how law determines the shape and the affordances of economic markets and how these markets define the power relations between different players in the RecSyss ecosystem. This will allow me to trace the consequences for the implied incentive structure. After thus sensitizing the reader to the impact of economic incentives I will discuss “the business and the math of persuasion,” starting with Packard's seminal description of “motivational research” in the 1970s, following up with current persuasion techniques and the design choices they involve. This will feed into a brief recall of the “behaviorist assumptions of micro-targeting,” as discussed in other work (Hildebrandt, 2017), focusing on old and new types of behaviorism and how they have given rise to dark patterns (Seaver, 2019). This directly relates to the issue of proxies, i.e., the machine-readable variables that stand for relevant features and targets.

Instead of reinforcing the belief that micro-targeting works as claimed, I will pause for an “intermezzo on counter profiling,” a technique that aims to infer how RecSyss apparently profile their end-users, thus enabling profiling of the profilers. I then discuss “human-machine feedback loops and the Goodhart effect,” inquiring into the pitfalls of cybernetic control over human behavior, explaining the notion of the Goodhart effect and how this relates to the kind of adaptive anticipation that is key to human interaction. Thus, I will trace the consequences of the Goodhart effect for both science and society. I conclude that developers of RecSyss must face the music and wake up from their behaviorist dreams, if they want to remain relevant in a world that is swiftly moving toward lifting the veil from unsubstantiated claims. The suggestion that developers may be prone to “behaviorist dreams” is not meant to shame individual persons but hopes to address the epistemic or interpretive community (Fish, 1980; Haas, 1992) of researchers that build RecSyss. In *The Return to Reason* Stephen Toulmin spoke of the pitfalls of “rationalist dreams” (Toulmin, 2003), arguing that the rationalist approach has failed on three fronts, refuting claims (1) as to a universal method, (2) a perfect language, while (3) reminding us that even within the natural sciences we cannot assume that nature offers us objective certainty. Part of Toulmin's argument returns in Cantwell Smith's recent work on the difference between reckoning and judgment (Smith, 2019) which celebrates the “unutterably rich metaphysical plenum” of real world objects, defending the concept of intelligence against the unworldly dreams of both good old fashioned AI (GOFAI) and AI's modern approach (AIMA). In this article I will argue that whereas GOFAI and AIMA have in a sense extended the “rich metaphysical plenum” (Smith, 2019) of our shared world, some of the beliefs underlying their application reverse the relationship between world and proxy in a way that diminishes our agency. Such a belief system (which inverses the relationship between a proxy and what it stands for) is what I refer to as built on “behaviorist dreams.” This also implies that asking me for “the data that proves my point” entirely misunderstands the point. As Gitelman, Cantwell Smith and many others have pointed out, data is not the same as what it represents, simulates, traces or signals (Gitelman, 2013; Smith, 2019) and this is why the issue of proxies is key here.

In the section on “the legal framework as a choice architecture” I will turn the tables on the usual narratives about “choice architecture” that focus on the choice architectures that RecSyss offer to enable nudging citizens and consumers. Instead, I will frame dedicated legislation as a choice architecture for those who provide or deploy RecSyss. I will explain how the GDPR and the AI Act build specific choice architectures for *controllers*, i.e., those who determine the purpose and means for processing personal data, and for *providers* of high-risk AI systems, i.e., those who develop (or have others develop) AI systems that they put on the market or put into service under their own name or brand.

In my conclusions, I will argue that both the GDPR and the AI Act will shape global economic markets in ways that will affect the design, default settings and interactive features of RecSyss. The type of constraints this entails will not obstruct research and innovation in RecSyss but instead foster and enable resilient, robust, reliable, and responsible RecSyss where end-users' agency is respected, based on user participation rather than relying on reductive user modeling. This is the most salient way to face the issue of proxies where it comes to human behavior.

## Defining RecSyss

As any handbook will tell (Jannach, 2010; Ricci et al., 2016) RecSyss are meant to offer relevant information in myriad settings, basically dealing with situations where an abundance of information makes it hard to detect and select what is required in a specific situation, or with situations where information itself cannot be accessed. Recommender systems are now part of search engines (the PageRank algorithm), streaming providers (music, movies, podcasts), social networks (rankings in timelines, news feeds), webshops (suggesting similar products or services), platforms that mediate the gig economy (recommending short stays or car rides), and will no doubt feed into cyberphysical infrastructures [internet of things (Felfernig, 2019), smart cities (Quijano-Sánchez et al., 2020), connected cars, smart energy grids (Chadoulos et al., 2020)]. On top of that behavioral advertising, which runs the business model of “free” services such as search engines and social networks, is a dedicated type of recommender system (Yun et al., 2020). On the one hand, behavioral advertising recommends products and services to potential consumers, on the other hand, it recommends advertising space to potential advertisers. In the current global marketplace, many recommender systems are in the business of persuasion, often combining advertising or marketing with offering relevant information.

It is crucially important that we acknowledge that without RecSyss neither individuals nor private or public organizations would be able to navigate, let alone address the current information overload. This, however, does not imply that anything goes, nor that we can simply refer to an agreed-upon objective standard of what information should be presented to whom, by whom, when, how and in what context. Technology is neither bad nor good, but never neutral (Kranzberg, 1986). In the next section, we will explore the incentive structures that currently drive the provision and deployment of RecSyss. Before that, we must clarify the backend of these systems and explain how its design informs their deployment and their impact on those targeted with their output.

Though I am using the term in a broader sense, RecSyss in a more narrow sense of the term are based on a relatively small set of filtering mechanisms that are tuned to specific types of users, content or both. These mechanisms involve inferences from behavioral data or input explicitly provided by users concerning their preferences. The main filtering techniques are collaborative based (inferences based on aggregate user behavior), content-based (retrieval of relevant documents in the context of a specific domain), or knowledge-based (often constraint-based, with input provided by domain experts) and various types of hybrids. This implies overlap with disciplines such as information retrieval, machine learning, symbolic reasoning, knowledge management, and search. As this special issue is focused on “human-machine feedback loops” we are reminded that human-machine-interfaces or human-machine-interaction (HMI) is another key discipline in this domain (Man, 2022).

Attention to human-machine interaction is often limited to end-user-friendly design and user modeling, hoping to achieve some goal as to the user's behavior. However, for RecSyss to operate in efficient and effective ways, keen attention must be paid to (1) the interaction between those designing and building RecSyss and the system under development, as well as to (2) the interaction between those who deploy the system to offer specific services and the system they use, and, finally, to (3) the interaction between end-users and the RS they engage with. Interactionist approaches that focus on the third type of interaction (and maybe the second), overlook that the first and the second actually determine the affordances of the system that end-users get to interact with; together, they define the *choice architecture* (Thaler et al., 2010) that controls what types of options the end-user has, thus constraining the kind of recommendations they will obtain. This invites a further inquiry into the economic incentive structures that keep developers, providers, and those who deploy RecSyss on their toes.

## A Political Economy of Recommender Systems

In 2008 we published *Profiling the European Citizen* (Hildebrandt and Gutwirth, 2008), co-authored by computer scientists, lawyers, social scientists and philosophers. We focused on the computational, legal, social, and underlying epistemological issues of using what was then called “knowledge discovery in databases” (KDD), inspired by early work on the complexities and potential impact of “group profiling” (Vedder, 1999; Custers, 2004). *Profiling the European Citizen* is still highly relevant in terms of issues such as bias, discrimination, privacy, and automation bias, but also pivotal as to a proper understanding of the manipulative impact of design and deployment of KDD and similar systems (such as RecSyss). The volume engaged a relational understanding of knowledge, as emphasized in the follow-up work of 2018, subtitled “*cogitas ergo sum*” (Bayamlioglu et al., 2018), acknowledging that the way others frame us is part of what shapes us. Recently, we have seen a number of best-selling works that make similar points about the impact of machine learning available in a more narrative style for a larger audience (Pasquale, 2016; Eubanks, 2018; Noble, 2018; Crawford, 2021), including the oft-cited work by Zuboff on “surveillance capitalism” (Zuboff, 2019). My goal in this article is not to repeat these points made, but to explain how law shapes economic markets (Cohen, 2019; Hildebrandt, 2019; Pistor, 2019) and how that relates to the issue of the proxies that RecSyss engage to make their models work. In other words, my goal is to show the importance and impact of the choice of proxies when developing RecSyss and to clarify why law matters for getting things right.

Law does not grow like grass, though it requires cultivation. Whereas, grass is an organism shaped by myriad biological interactions, law is an *institutional fact* (Anscombe, 1958; Searle, 1969; Austin, 1975) largely shaped by written legal *speech acts* whose performative effects “make” the law (MacCormick, 2007). Being text-driven and based on natural language ensures a combination of semantic continuity and adaptive flexibility that is core to law and the rule of law (Hildebrandt, 2020). The relationship between speech acts and the institutional facts they create is not causal but constitutional and the same goes for the institutional facts created by the law, such as economic markets. Law shapes the choice architecture offered by economic markets, both where it enables (contract law, tort law, property law) and where it constrains (private law, public law, human rights law). If contract law did not cover the legal effect of a lack of performance, we could never be sure about the future consequences of a contract, which might then come to depend on prevailing power relationships rather than on what parties agreed upon. This would dissolve the binding character of law that sustains economic markets. If property law would not provide right holders with broad powers to use, alienate, or even destroy their property, markets-as-we-know-them would not exist. If private law did not void contracts based on undue influence or fraud, markets would soon become dysfunctional or collapse into patronage systems. Those who care about democracy and the rule of law should pay keen attention to how markets are constituted, how they distribute powers and liabilities and thereby both wealth and inequality (Pistor, 2019).

Thus, economic markets do not grow like grass, but depend on how we create and sustain them by way of binding legal norms that in turn enable people to engage in binding agreements. This invites an inquiry into the kind of incentive structure that these markets themselves create, based on what complex interplay of economic power relationships is enabled. This is the objective of sketching the political economy of recommender systems, i.e., figuring out what drives their provision and use; what players are enabled or kept in the dark.

Once we can trace the economic power relationships, we can inquire into the way they influence the methodological integrity of these systems and their reliability in terms of the functionalities claimed for them. This will also allow us to test how the GDPR and the proposed AI Act may contribute to an incentive structure that favors dependable systems and responsible deployment. Different sets of legal norms create different types of markets; economic markets are not given but constituted, they can be reconstituted if we want to. In this article I do not aim to provide a granular analysis of specific RecSyss, but rather to develop the framework to situate economic power relationships and incentive structures. The point of the article is to clarify how these incentives impact upstream design decisions, in particular concerning the choice of proxies.

## The Business and the Math of Persuasion

### Hidden Persuaders Old and New

To understand the political economy of recommender systems we must focus on the power relationships between different players. These are (1) providers of products or services, e.g., advertisers, energy suppliers, webshops, (2) publishers that host recommendations, e.g., websites, search engines and social media that sell space for advertising, (3) intermediaries that negotiate between publishers and providers and/or between publishers and consumers, and (4) consumers or end-users targeted with supposedly or hopefully relevant recommendations.

In the context of the political economy of recommender systems the question of relevance is crucial. Relevance is a key concept in information retrieval (Froehlich, 1994; Saracevic, 2007) and in the design of recommendation systems (Jannach, 2010; Ricci et al., 2016). The key question, however, is relevant-for-whom: for the consumer or end-user? for those paying the bill, e.g., an advertiser? or for those making a profit, such as the publisher and the intermediary? In a market that thrives on free services it makes sense to check who pays what bill in exchange for what service, and even in a market where consumers do get to pay the bill (subscriptions) it makes sense to figure out what drives the recommendation (user preferences or user nudging; Zou et al., 2019).

In 1957, Vance Packard described the cynical use of pseudo-scientific insights in marketing and advertising, in his seminal *Hidden Persuaders* (Packard and Miller, 2007). In this work he revealed to what extent advertising and marketing were based on defunct “insights” of commercially operating psychoanalysts, behaviorists and other folk, “sold” under the heading of “motivational research” (MR). MR was mostly grounded in unsubstantiated claims about how to manipulate the irrational unconscious of consumers or citizens (voters). In fact, MR was mainly deployed to persuade advertisers to buy into pseudo-scientific claims about how to persuade people to purchase what they may neither need nor want.

The conflation of machine learning and nudge theory that is the focus of this article, seems a direct heir of MR, based on similarly untested claims regarding the effectiveness of behavioral targeting. And, once again, the success must be located in persuading the advertisers rather than voters or consumers. This, however, does not imply that the use of recommender systems makes no difference. As Packard notes in his final chapter on “The Question of Morality,” the aim to manipulate rather than inform people has moral implications:

What are the implications of all this persuasion in terms of our existing morality? What does it mean for the national morality to have so many powerfully influential people taking a manipulative attitude toward our society? Some of these persuaders, in their energetic endeavors to sway our actions, seem to fall unwittingly into the attitude that man exists to be manipulated.

Mapping the political economy of recommender systems aims to unearth the perverse incentives that drive systems supposedly meant to *infer* preferences. Actually, these systems are *rewarded* for influencing these preferences while catering to them. There is a loop here that keeps those targeted in an echo chamber, for instance *reinforcing* whatever elicits further “user engagement” (Zou et al., 2019). It seems key here to differentiate between online behavioral advertising run by big tech platforms, such as Google Adword, and, for instance, music or movie recommendations run by dedicated service providers, such as Spotify or Netflix. Depending on the extent to which providers maintain quasi-monopolies in the relevant market, the implications of skewed incentives will differ (Hildebrandt, 2018). Not because users will simply choose another provider if they can but because the ability to influence the users' choice architecture increases due to network effects and their role as gatekeepers of relevant information. In other words: users will have no inkling what recommendations they miss. The point of this article is to demonstrate that insofar as behaviorist assumptions underpin these systems, they are all prone to the same perverse incentives, in part “making” the preferences they claim to “mine.”

### Design Choices: The Issue of Proxies

The techniques of *collaborative filtering* that underly many currently deployed RecSyss assume several design choices that determine the output of RecSyss. First, the purpose must be specified as a task, which necessitates a formalization that serves as a proxy for the “real” purpose. The real purpose may be providing an end-user with what they like/need/want (already three very different concepts). Formalization will probably be done by (1) stipulating that the system is given input data about what items/services/opinions they engaged with previously (where “engaged” will require formalization in terms of, e.g., click or purchasing behavior) or (2) stipulating that the system is given input data about what items/services/opinions similar end-users engaged with (which entails formalization of “similar end-users”). The proxies involved are the measurable historical behavioral data of the end-user (e.g., their click- surf- or purchasing behavior) and/or measurable historical behavioral data of similar end-users. Though it may be very interesting to make inferences and provide recommendations on this basis, one can debate whether an end-user's liking/needs/wants can be reduced to such input. Another way of framing this is asking whether the developers are doing a good job in “groundtruthing” (Campagner et al., 2021), i.e., in providing the learner algorithm with the right model for what would be a correct output, where that model is basically the distribution of the data on which the learner is trained, validated, and tested. With an eye to methodological integrity, it is important to acknowledge that we have here a prime example of the use of a measure (the distribution of relevant data) as a target (steering end-users toward a similar distribution in the future). I will return to this under “human-machine feedback loops and the Goodhart effect.”

One might counter that click-, surf- or purchasing behaviors are themselves real-world features, not proxies. This would mean that as to features, the problem can be solved by moving from labeling to “direct input.” However, the datafication that takes place, turning dedicated measurements into specific variables, framed as “raw data” (Gitelman, 2013), is then hidden under a kind of “naturalization” that erases the difference between human action on the one hand and its transformation into discrete measurable behaviors on the other. This is the main thrust of Cantwell Smith's argument in *The Promise of Artificial Intelligence* (Smith, 2019), highlighting that computer data is not the same as real world objects, states, or actions.

Though proxies inevitably skew the output, this may have perverse consequences in the case of the RecSyss that are the target of this paper, as they will include proxies for persuasion-oriented goals that will inevitably influence the output (=recommendations). Again, almost by definition, the system will be using a measure (the distribution of relevant data) as a target (steering the end-user toward a similar distribution in the future). The point here is that, depending on the goals, “the distribution of relevant data” will be compressed into different algorithms, resulting in different recommendations; “the” distribution does not exist, unless in the form of replicating the entire data set (Hildebrandt, 2021a).

We may conclude that big players who develop and/or provide RecSyss have a clear incentive to combine the purpose of providing relevant recommendations with persuasion purposes, meant to increase, e.g., advertising revenue or to *prime for* specific types of political opinions, food choices or lifestyle behaviors.

## Behaviorist Assumptions of Micro-Targeting

### Behaviorism Old and New: The Inversion of the Proxy Relation

Behaviorism is a way of “doing” science that goes back to Pavlov, Skinner and Watson and informs both behavioral economics (nudge theory) and its “reception” in computer science. In other work I have traced the problematic assumptions of behaviorism and their implications for the output of machine learning applications in learning analytics (Hildebrandt, 2017). These assumptions regard the fact that behaviorism often inverses the relationship between concepts and their proxies, based on a belief that animal and human agency consists of discrete, observable, measurable units of behavior (e.g., producing saliva, ticking boxes on a screen, “firing of neurons”) which are imprecisely expressed in concepts such as appetite, consent, thinking. This assumption is a bug from the perspective of scientific inquiry, because it confuses the modest proposition that we only have access to what we can observe with the claim that only that which we can observe exists and/or matters^5^. From the perspective of developers of RecSyss this—flawed—assumption may seem to be a feature, because it promises more precise and accurate knowledge due to the fact that supposedly vague concepts can be replaced by the behavioral primitives that inform them.

The problem of turning the relationship between proxies and concepts inside out was key to the so-called *Methodenstreit* that raged in economics and social science during the first half of the twentieth century, with spill-over into the late twentieth century “science wars” (Hauser, 1988; MacLachlan, 2017; Stadler, 2020). The kind of behaviorism I am referring to underlies most work in econometrics and defines the point of departure of both Chicago Schools (rational choice theory as well as nudge theory). They both depend on what has been called “methodological atomism” (Heath, 2020), a way of framing reality as an aggregate of discrete datapoints rather than, e.g., acknowledging that a training dataset is nothing but a proxy for the “truth” we want an algorithm to learn. The difference is that rational choice theory (classical economics) assumes that the atoms are rational and therefor predictable, whereas behavioral economics (nudge theory) assumes that the atoms are irrational but nevertheless predictable (Ariely, 2011).

Micro targeting (MT) is core to the personalized recommender systems we discuss here, that is those intended to serve two masters: the end-user and the service provider. MT that is based on collaborative filtering requires hoarding of behavioral data of end-users (their own historical data and/or those of their similes). It is easier to “sell” this with the hidden assumption that such data are the primitives that disclose our deepest intentions, desires, and vulnerabilities. From a scientific perspective, however, it is important to acknowledge that these primitives—as well as any inferences based on them—are mere proxies for what the provider aims to know about the end-user. Those working with RecSyss based on MT are probably aware of this (Frederik and Martijn, 2020), but it is not in their interest to acknowledge this as it would probably deflate the business model that funds them.

### Dark Patterns

If the economic incentive structure invites business models that accept bugs as features, we should not be surprised that “dark patterns” abound (Seaver, 2019), aiming to sell RecSyss as innovative ways to influence end-users in a direction they might otherwise not have chosen. Though this may be qualified as manipulation, neoliberal ideology will present this as a way to grow the economy that is beneficial for those manipulated^6^, while public policy pundits will portray this as benevolent paternalism (Sunstein, 2016). Here the concept of a choice architecture is pivotal. “Choice architecture” is a term of trade in nudge theory (Thaler et al., 2010), referring to the types of choices that are offered to end-users, as consumers of goods, political opinion or whatever else. Choice architecture refers to a built environment (stones or software) that favors some choices over others, thus “nudging” folk into the preferred behaviors of policy makers, webshops, or advertising intermediaries. If it pays to lure consumers into dedicated behavior patterns, preferably without their awareness, and if this is not prohibited, those with the means to do so may in some sense be forced to engage in such dark patterns as they believe they could otherwise be pushed out of the market. We should, therefore, not be surprised that “dark patterns” of subliminal manipulation have indeed emerged.

What is more interesting is that some of the parties that play a key role in the political economy of RecSyss are pulling out, ignoring behavioral profiling in favor of, e.g., contextual advertising. The New York Times stopped its behavioral advertising in Europe when the GDPR came into force and reported an increase in advertising revenue (Davies, 2019). Before that, Proctor and Gamble substantially reduced its digital advertising budget, reporting a 10% increase in outreach 1 year later (Johnson, 2018). Doubts are being cast on the actual effectiveness of behavioral profiling (Lomas, 2019; Masnick, 2021), papers are written on the limited gains for publishers (Marotta et al., 2022) and on the pervasive misalignment of the economic and other incentives for different players (Marotta et al., 2022). My point here is not the moral and political argument against surveillance-based advertising (The Norwegian Consumer Council, 2021), but a more foundational issue about the fault lines in the narrative about its effectiveness and reliability. Though it is crucial to make those moral and political arguments against manipulation, it is perhaps even more critical to better understand both the potential and the limits of micro targeting.

## Intermezzo on Counter Profiling

In 2020, Facebook served researchers from the NYU Ad Observatory^7^ with a cease-and-desist letter, threatening legal action if they would not stop collecting targeting data regarding political ads (Facebook, 2021; Watzman, 2021). Apart from closing the accounts of the researchers, Facebook also disabled application program interfaces (APIs) that allowed Facebook users to donate their data to the Ad Observatory to compare how people are actually targeted with political ads with the Facebook Ad Library that supposedly provides transparency about their targeting practices. The Ad Observatory found that many ads were missing from the Facebook Ad Library and tried to profile how Facebook's algorithms decide to target ads, something that Facebook does not disclose. In 2021, Facebook made changes to its website code that make it more difficult to identify sponsored posts, thus further frustrating efforts by independent researchers to scrutinize the way political advertising actually works on Facebook (Faife, 2021). The Observatory basically engages in what I once coined counter-profiling, or profiling the profilers (Hildebrandt, 2009, 2015).

In a report on behavioral biometric profiling from 2009, we distinguished between two types of transparency enhancing tools or TETs (Hildebrandt, 2009):

Type A: legal and technological instruments that provide (a right of) access to (or information about) data processing, implying a transfer of knowledge from data controller to data subjects, and/or

Type B: legal and technological instruments that (provide a right to) counter profile the smart environment in order to “guess” how one's data match relevant group profiles that may affect one's risks and opportunities, implying that the observable and machine readable behavior of one's environment provides enough information to anticipate the implications of one's behavior.

Whereas, type A depends on the willingness of a company or other entity to provide information about or access to relevant data (or on an effective right thereto), and on the technical ability to check whether this information is correct and/or the data complete, type B simply applies the same techniques of data-driven profiling that online and offline environments engage, to counter-profile that same environment. In other research, the DataBait tool^8^ was developed with a similar objective. Based on data-donations by users of social networks, we hoped to provide them with the kind of profiles that could be drawn about them, with a range of sensitive attributes (Popescu, 2016). Though the tool was not operational by the time the project finished, it might have generated similar attention from Facebook if it had been successful.

In 2021, Algorithm Watch announced that Facebook had threatened them with legal action if they continued their research on the Instagram Newsfeed algorithm, which Facebook found in violation of its Terms of Service (ToS) and the GDPR. Though both claims seem debatable if not far-fetched, Algorithm Watch decided to halt its research stating it does not have the means to start a legal battle with a tech giant. Note that the ToS prohibits scraping data from Facebook products with automated means, whereas Algorithm Watch only collected data from Facebook users who installed a plug-in to share what ads they receive. Note also that any content from other users was immediately and automatically removed, to be in compliance with data protection law (as these other users had not given their consent). Algorithm Watch then addressed an Open Letter to Members of the European Parliament, pressing for legislation that should enable this type of public interest research, more notably asserting the need to uphold and refine art. 31 of the proposed Digital Services Act (DSA)^9^ that gives “vetted researchers” with academic affiliations access to platform data for public interest research^10^.

In point of fact art. 29 and 30 of the DSA require transparency regarding parameters and other relevant data whenever recommender systems and online advertising are used by very large online platforms. Moreover, art. 29.1 DSA stipulates that the providers of these platforms must clarify in their terms of service:

any options for the recipients of the service to modify or influence those main parameters that they may have made available, including at least one option which is not based on profiling as defined in Article 4 (4) of Regulation (EU) 2016/679. Providers of very large online platforms shall also make this information directly and easily accessible on the specific section of the online interface where the information is being prioritized according to the recommender system.

Counter profiling, however, is not equivalent with providers having to give users a chance to modify or influence a parameter. Rather, counter profiling (1) enables a better understanding of whether recommender systems deliver what they are claimed to deliver and (2) it can trace some of the actual consequences of microtargeting, especially at the aggregate level. It might enable us to demonstrate how the entire information ecosystem is distorted in the process of feeding targeted individuals humbug, even if those individuals have learnt to resist and avoid manipulation^11^.

## Human-Machine Feedback Loops and the Goodhart Effect

### Illusions of Control Over Human Behavior: The Return of the Proxy

The interesting question is why Facebook gets so upset when their targeting data are scrutinized from the outside, that is from the perspective of their users—based on the same types of techniques they deploy to target them. The Ad Observatory found that the Facebook Ad Library (a repository of relevant data made available by Facebook) was incomplete in ways that contradict the claims made by Facebook. It seems the repository is carefully curated to prevent anybody from gaming the Facebook algorithms and/or from figuring out the real-world (in)effectiveness of their behavioral profiling. Returning to the current incentive structure (as shaped by relevant legal architectures), we could simply conclude that “the problem with Facebook is Facebook,” evidenced by, e.g., the work of Vaidhyanathan (2018) and inside testimonies that lift the veil of Facebook's hidden agendas (Roose, 2021). My aim here is, however, another one. I believe that more is at stake than dirty politics within big tech, enabled by inadequate legislation or spineless enforcement. The point is also that the underlying belief in our ability to manipulate human “behaviors” (calculable changes in the state of human beings) is deeply flawed, mistaking proxies for what they are meant to represent.

To understand the issues at stake at a deeper—and simultaneously highly practical—level, we must pay keen attention to a crucial caveat that haunts all attempts to control human behavior based on the measurement of discrete behaviors. Measurement of human interaction requires quantification, but quantification paradoxically requires prior qualification (Callon and John, 2005). To measure “how many people like long hair” one has to qualify certain behaviors as “liking” and certain hair as long hair, which will involve all kinds of shortcuts or proxies. In times of Facebook, “liking” may refer to clicking a thumbs-up icon, but that's a very remote proxy for people actually liking something. Long hair may refer to certain dog breeds, to women or to men, to various types of hair (we do not only have hair on our heads, mind you). You may notice that specifying the kind of hair intended is less cumbersome than specifying what we mean with “liking.” Some concepts are more rich, depending on a more complex type of tacit knowledge (Polanyi, 1966), while such concepts are often what Gallie called “essentially contested” (Gallie, 1956), meaning that there is fundamental disagreement on what they refer to. Resolving that disagreement by singling out one meaning and imposing it on the research design to solve the engineering problem may result in skewed output. In short, whatever formalizable and quantifiable proxy one selects for “liking,” the quantification will always (1) require an act of qualification of concrete instances as “liking” (whether that qualification is done by hand or automated) and (2) involve opting for one interpretation over others, thereby *loosing* the kind of tacit knowledge that grounds our understanding of “liking” while *gaining* computability. In the case of supervised machine learning this is visible in the need to pay for a cohort of laborers to label (qualify) the training data, hiding the act of interpretation that is involved in this qualification^12^. On top of that, those who instruct the laborers impose their qualification on the training data, which will constrain the potential output of the learning algorithm as it can only see what has been labeled (framed) as “liking.”

As indicated above, RecSyss that seek to offer what consumers “like” require human-system interaction on three levels, that of (1) development, (2) deployment, and (3) micro-targeting. Interestingly, the interaction between developers and the system under construction will impose constraints on what the system is capable as recognizing as “liked” items (whether an opinion or a product or service). The same goes for the interaction between whoever deploys the system, as they will choose certain default settings, thus further shaping the choice architecture of the end-user (who will receive some recommendations at the cost of others). At the level of the design of the system certain behaviors will be used as a measure of what users like, and because that measure is then used as a target the targeted human will end up in a feedback loop that keeps them in an echo chamber or filter bubble. The filters that are created at the level of design and deployment turn into a rather boring menu of recycled preferences (the preferred term for “likes”). Because human beings are whimsical, ingenious, and forever anticipating how others anticipate them they will stop “liking” what they have been foreseen to “like” and behave in ways the system may not capture because it cannot see outside the qualifications that trained its algorithms (even if these qualifications were inferred based on unsupervised learning).

### Goodhart Effect

In economics this has been identified in 1975 by Goodhart as the adage that (Goodhart, 1984)^13^:

Any observed statistical regularity will tend to collapse once pressure is placed upon it for control purposes.

In later work by Strathern this has been summed up as “the Goodhart effect” (Strathern, 1997):

When a measure becomes a target, it ceases to be a good measure.

The reason for this is simply that human agents will respond to the use of the measure with a change in behavior that invalidates the measurement as such. This goes for the behavior of those using the measure as a target, and the behavior of those targeted. The measurement therefor keeps lagging behind what it aims to measure, while probably achieving numerous unintended side-effects. Strathern demonstrated this in her seminal article on the use of quantitative indicators for the quality of higher education, for instance, the number of students that enroll in a university, obtain their diploma, find work after graduating or, on the side of research, the number of publications, citations and journal impact. Obviously, once researchers are aware that their livelihood will depend on their measurable output they will change their expectations as to what counts as quality and change their behavior to ensure a strong “track record,” while the management layer will change its assessment of quality research from one based on skilled intuition and content to one based on quantifiers and metadata. This is not a matter of researchers trying to game the system but rather a matter of them becoming part of a feedback loop that invites all kinds of “elephant paths” to achieve the goals set for them (e.g., publishing the same content with slight tweaks in different journals, claiming novelty when merely repeating state of the art). This need not be a matter of deliberate deception but will rather be an intuitive change of direction that becomes the new common sense, resulting in new journals to accept the growing number of articles, infeasibility to read all that is published, in turn resulting in increased dependence on citation metrics. This is where the circle closes; the measure of high quality (high impact) becomes the target (publishing in high impact journals), driving a competitive “market” for publications that define career paths. In other words: while the proxy of the target is mistaken for the target itself, the aim of high quality research turns into the aim to provide observable, quantifiable evidence of high quality. Again, the proxy is taken for what it stands for, inversing the relationship along the lines of good old behaviorism.

As we are restricting ourselves to systems that seek to *influence* what consumers “like” *while mining* what they “like,” the Goodhart effect will play out in even more perverse ways than it would if the only target were to provide people with their inferred preferences. A retailer offering the right product to a customer may be successful, an advertiser creating a need/desire/want for a particular product may be equally effective, but trying to simultaneously create, predict and target individual preferences on a massive scale will not work in the long run. Not because recommender systems are not sufficiently sophisticated, e.g., based on deep learning techniques testing ever more parameters resulting in mathematically precise personalized recommendations. On the contrary, whatever mathematical precision is achieved, it will forever be off the mark as people intuitively anticipate how they are profiled, shift their expectations, and change their interactions. This also implies that “fixing” the Goodhart effect by way of better metrics, as some suggest (Stray, 2021), may instead exacerbate rather than solve the problem.

### Adaptive Anticipation

The anticipation I refer to is not based on the kind of calculation that is the hallmark of rational choice theory, but rather on the intuitive learning capacity that some hold to be “irrational but predictable.” Instead of framing this intuition as irrational (wrongly biased), as Kahneman does (Kahneman, 2003), we can better understand this anticipation as based on our ability to deal with complexity by way of smart heuristics (rules of thumb) that are developed in tune with our situated or ecological rationality, as Gigerenzer argues (Todd and Gigerenzer, 2007).

Instead of framing our rationality or irrationality as properties of atomistic individuals, human rationality should be understood as a relational “thing” that hinges on human interaction within a particular environment. Rational choice theory's assumption that human beings are optimization machines hoping to maximize their own interests/preferences/benefits, can be understood as part of a utilitarian ideology that similarly underpins behavioral economics' nudge theory. The only difference between the two is the latter's caveat that humans suffer from cognitive biases that distort their ability to make the right choice, which is nevertheless the same type of rational choice that informs the other Chicago School. Based on a specific interpretation of Darwin, this ideological portrayal of individual human beings as intent on optimization of their own interest at the cost of others has been “naturalized” as a given biological fact (Favini, 2020), often invoking hunting and gathering societies as forever determining our competitive-aggressive nature^14^. Human beings, however, are far too complex, situated and adaptive to be reduced to *homo economicus*, noting that culture, shared values and normative frameworks are dynamic and forever on the move.

The Goodhart effect, then, is not caused by folk trying to game the system but by interacting people that—while also interacting with RecSyss—dynamically intuit how they are being framed with what potential consequences; they will continuously reconfigure their responses accordingly. Such reconfiguration does not depend on them correctly intuiting how they have been framed but it does imply that the distribution of historical behavioral data will be different from that of future data. The most fundamental assumption of the kind of machine learning that drives many RecSyss, namely that these distributions are similar (Mitchell, 1997; Hildebrandt, 2021a), is wrong where it concerns human behavior. In machine learning terms: historical data is not necessarily a good proxy for future data. Whoever build their castle on the proxy without taking this into account, are building on sand.

### Methodological Integrity in Science and Society

The bottom-line of the previous sections is that the combination of behaviorism, machine learning and nudge theory will not deliver what it promises while nevertheless creating havoc. The foundation of this toxic cocktail of (1) hidden behaviorist assumptions, (2) mistaking a proxy for the thing it supposedly stands for, and (3) automating attempts to manipulate user engagement, reconfigures the private and the public sphere based on what is now euphemistically called “misinformation.” From the perspective of computer science we thus face a detrimental lack of methodological integrity, due to the deployment of mathy pseudo-science (Brooks, 2017), or—a more modest claim—mistaking exploratory research design for confirmed output (Hofman et al., 2017), or—the internal critique—an incentive structure that invites ML papers that focus on algebra instead of new insights (Lipton and Steinhardt, 2019).

In the meantime, this is not just about the discipline of machine learning. This is about how we navigate the real world, because in the end that is what RecSyss aim to do: to improve our capabilities for navigating our physical and social environment in real life. To achieve methodological integrity within science in order to protect society against the deleterious effects of pseudo-scientific predictions we need to change human-machine-interaction at the level of the providers of RecSyss and of those who deploy them. This will require changing their choice architecture, to make sure the end-users get the recommendations they need/like/want in a way that respects their agency.

## The Legal Framework as a Choice Architecture

### Hijacking the Concept of “Choice Architecture”

Nudge theory has invented the concept of a choice architecture, to frame a deliberately designed environment that determines the types of choices available. The objective of creating a particular choice architecture is to exploit one or more cognitive biases to lure human agents into whatever behavior is preferred by the provider. Nudge theory clarifies that RecSyss are designed to not only retrieve but also to influence end-user preferences. In the US, nudge theory and choice architecture have been embraced by public policy gurus such as Cass Sunstein, who argues that public policy should make use of them to help citizens to make the right choices (Sunstein, 2016). This is qualified as libertarian paternalism, justified by the need to correct supposedly predictable irrational bias in human decision making. As discussed above, nudge theory is built on sand. I believe, however, that the concept of a choice architecture becomes very interesting if we liberate it from behaviorist assumptions and from attempts to influence people “behind their back,” instead of having them participate in the design of their environment. One could even say that this is what democracy is all about (Dewey, 1927): making sure those who suffer or enjoy the consequences of the built environment have a say in how this is done, while noting that “built” refers to both our physical and our institutional environments.

Freed from naïve interpretations of human agency we can connect the concept of a “choice architecture” with the concepts of “affordance” and “capability” that both highlight the relationship between a human agent and their environment. Together, affordance theory (Gibson, 1986; Heras-Escribano, 2019)^15^ and capability theory (Sen, 1999; Robeyns, 2005) understand agents in terms of what an environment “affords” them and of what affordances they are “capable” of acting upon. From that, ecological and relational, perspective, the notion of a choice architecture refers to what a particular built/designed/institutional environment affords specific types of agents in terms of the types of choices they can and cannot make, depending on their capabilities. This refurbishing of “choice architecture” enables keen attention to how affordances depend on agents and vice versa—while also taking note that much of our environment is not built in stone but in terms of what has been called institutional facts.

A proper understanding of the notion of “institutional facts” is key to understanding choice architectures in real life, as opposed to those being designed and tested in controlled environments. Institutional facts are facts created by performative speech acts; that is speech acts that “do” what they “say,” where speaking is acting. The seminal example is the civil servant declaring a couple husband and wife, thus instituting their marriage, with all the legal effects attributed by the law of the land to the status of a marriage. The civil servant does not “cause” them to be married, nor “influenced” them into concluding the marriage, the declaration actually marries them.

Legal frameworks such as data protection law (GDPR) (see text footnote 3) and the proposed regulation for AI systems (AI Act) (see text footnote 4) create a choice architecture for controllers and processors who process personal data (GDPR) and for providers and users of AI systems (AI Act). This legal choice architecture affords them a dedicated set of choices on how to design, further develop and deploy their systems. The legislation aims for legal protection by design and default (Hildebrandt and Tielemans, 2013), thus requiring specific choice architectures to be engineered to empower end-users. The law itself, however, consists of institutional facts that are constituted by the written legal speech acts of the European legislature, articulated in natural language and thus explicit, multi-interpretable by default and therefor contestable. The purpose of the law is not to nudge folk behind their back into behaviors that legislatures prefer for them. The aim is to make the legal norms that a democracy has agreed upon explicit, enforceable and simultaneously contestable. The law and the rule of law thereby respect and appeal to human agency in a way that persuasive RecSyss do not. This raises the fascinating question of what “legal protection by design” could be.

### The Choice Architecture for Controllers Under the GDPR

As discussed above, economic markets do not grow like grass, they are not brute facts but complex institutional facts with far reaching performative effects that co-determine the choice architecture faced by users and end-users of RecSyss such as corporations, consumers, employers and employees, house owners, tax payers, those who seek employment as well as government agencies, energy suppliers and big tech platforms.

The internal market of the EU is a prime example of a market that is created, enabled, and constrained by private and public law of the member states and by myriad legal instruments enacted by the EU legislature and further developed by the Court of Justice of the EU (CJEU). More specifically, the choice architecture of those who process personal data is determined by the Charter of Fundamental Rights of the EU, the GDPR, and other applicable legislative instruments, the relevant case law of the CJEU and other relevant sources of law (notably the Opinions of the European Data Protection Board and the European Data Protection Supervisor), together coined as the *EU data protection acquis*. For all corporations wishing to compete on the internal market of the EU, this acquis has relevance, even for those operating from outside the EU. This has been coined the Brussels effect (Bradford, 2012; Bygrave, 2021), highlighting how EU law shapes the global incentive structure for and of economic markets.

In this section, I will select the most salient elements of the choice architecture of the EU data protection acquis insofar as relevant for RecSyss. The ultimate goal is to emphasize how this will also affect the design of RecSyss' backend, and transform the way developers, providers, and users of RecSyss interact with them.

Current RecSyss are often based on the collection of online and possibly offline behavioral data [e.g., clickstream and surf behavior, purchasing behavior, location, traffic and navigation data, and data collected via quick response (QR) codes]. The first thing to note here is that the data that is collected are considered personal data if the related person can “reasonably likely” be identified. Due to the linkability of much behavioral data with other data this will often be the case (Brasher, 2018)^16^, triggering the applicability of the GDPR. Once the GDPR applies, those who determine the purpose and means of processing become liable for complying with the GDPR, which will mostly be the user of the system (not being the end-user but e.g., a webshop or the provider of a social network or search engine). This entity is called “the controller”^17^, and they have to ensure that they have a legal basis for the processing, which may be consent or a legitimate interest^18^. Neither can be taken for granted, as the requirements for informed consent are high^19^ and data subjects (those whose data is processed) must be able to withdraw their consent at any time in a way that is as easy as when they gave their consent^20^. The legitimate interest of the controller can only be used as a legal basis if it is not overruled by the interests, rights, and freedoms of the data subject^21^, noting the data subject has a right to object to the processing notably when based on the controller's legitimate interest^22^. This may sound dry and boring, reminding the reader of consent banners that pop up when surfing the web, disturbing the seamless “user experience” of the end-user, in turn disrupting the seamless and subliminal influencing mechanisms that drive the business models of the users of RecSyss^23^. Though it is tempting to frame these legal requirements as obstructions, they may in fact enhance end-user agency. The sand that is thrown into the machine of smooth intuitive interactions *creates a different choice architecture* that forces all parties to pay keen attention to their different roles and reminds them of the fact that sharing data is a choice with consequences. As those consequences should be clear for end-users, cookie banners could be an example of what some have called “apparency” (Schraefel et al., 2020) or “actionable transparency,” and to the extent that banners slow down both surfing and tracking it may be an example of what Paul Ohm called “desirable inefficiency” (Ohm, 2020). In saying “should be clear” and “could be an example” I wish to highlight that the choice architecture of the GDPR has *force of law* not *force of technology*; it does not preclude disobedience and much will depend on (1) the extent to which legal norms are internalized as the right way to act and on (2) the way legal norms are enforced [noting (1) and (2) interact]. The transparency requirements imposed by the GDPR nevertheless reconfigure the choice architecture of controllers who should be open as well as specific about their purposes to be lawful^24^. This entails that they should own up to the fact that their recommendations have the specific purpose of increasing their own revenue, which will allow end-users to foresee potential bias that is inherent in the recommendation.

This brings us to the purpose limitation principle. The requirement of necessity that is core to the GDPR's purpose limitation principle is perhaps even more important than the legal basis. The purpose limitation principle restricts all processing to what is necessary for the specified purpose^25^, noting that the principle obliges controllers to define one or more legitimate purposes and to make them explicit. In line with that, consent is only valid if provided for a specific purpose and if the processing for which consent is given is necessary for that purpose^26^. This is related to the data minimization and storage limitation principles that *configure* the controller's *choice architecture* in a similar way: they are bound to choose processing operations that are not merely appropriate but necessary for the intended purposes. This implies keen attention to what data is necessary to provide relevant recommendations and—as indicated above—it would require those deploying an RS to be explicit as well as specific about additional purposes that factually determine what and how data is processed or further processed. I could imagine that consent is freely given for the purpose of providing the end-user with relevant recommendations, whereas consent to provide recommendations that are foremost in the interest of the service provider, advertiser, political party or tech platform will most probably not be given, unless based on undue influence (e.g., dark patterns, or making the provision of a free service dependent on consent for additional processing)^27^.

There are more and other elements in the GDPR that co-determine the choice architecture that defines the market for RecSyss within EU jurisdiction, and thus also impacts design decisions for RecSyss that configure the backend system that end-users cannot access or control. For example, we can look into the prohibition of fully automated decisions that have legal or similarly significant effect on the end-user^28^. There is no case law on this particular element of the GDPR from the CJEU, but some relevant case law at the national level. The prohibition has three exceptions: consent, a legal obligation or contract. Those exceptions, however, are conditional upon certain safeguards, one of which is the obligation to provide meaningful information about the logic of processing^29^ which has caused a flurry of research into what some have called “explainable AI” (Edwards and Veale, 2017; Xu et al., 2019). Here again we see that *the choice architecture* for providers and users of RecSyss is reconfigured by such obligations, prompting providers to require that developers ensure that decisions taken by an RS are explainable to those targeted. One of the crucial questions here is what constitutes a “decision” in the sense of the GDPR, taking note that both during the design and during the deployment of a RecSyss, many technical decisions are taken fully automatically by a system that may have a significant impact on end-users (Binns and Veale, 2021).

Law, economic markets and RecSyss interact in myriad ways. Considering the perverse effects of deploying RecSyss that are dependent on behavioral data (see above on the Goodhart effect) and the even more detrimental effects of optimizing recommendations in view of the interests of those who deploy the system rather than the end-users, we should be grateful for the choice architecture that is put forward by the EU data protection acquis, more specifically the GDPR. It is not so much an obstruction of seamless user experiences and innovative service provision, but instead an incentive to pay keen attention to the methodological integrity of developing RecSyss. Do they serve the claimed intended purpose? Is the processing of personal data necessary for that purpose? The GDPR contributes to “good” innovation rather than innovation “*per se*” and it may thus help to break the vicious circle of perverse feedback loops. This will hopefully contribute to new ways of developing RecSyss, based on end-user *participation* instead of end-user *modeling*. This is one of the key points of Ekstrand and Willemsen (2016), raising a number of important questions as to what should count as meaningful participation that respects the agency of those invited to engage. This involves both methodological issues in the context of social science and philosophical inquiry into the nature of human agency and what it requires (Mekler and Hornbæk, 2016; Lyngs et al., 2018; Chevalier and Buckles, 2019; DeJonckheere et al., 2019). This article is focused on the preconditions of developing human agency and on the role of law in ensuring that RecSyss do not engage in a subliminal reconfiguration of what we need/desire/want. In that sense the current inquiry precedes empirical research that may actually assume what should be investigated, such as the “fact” that people have given preferences or that policy makers generally know what is best for people's health or well-being (Sunstein, 2016).

### The Choice Architecture for Providers Under the AI Act

The EU AI Act (AIA) that was proposed in 2021 is still under consultation and negotiation while finalizing this paper. As the final text is not yet available, I will restrict myself to highlighting the groundbreaking nature of the Act, the choices made when defining AI and the kind of requirements facing providers of high risk AI systems. Though the current proposal is not perfect in any sense (see my feedback to the Commission (Hildebrandt, 2021b)), the analysis below should illustrate how the Act may reconfigure the choice architecture of the providers of what the Act calls “AI systems.” As to its actual effectiveness much will depend on the budget allocation to national supervisors and on the upcoming revision of the EU Product Liability Directive which may impose strict liability on providers of high-risk RecSyss^30^, especially if their Conformity Assessment turns out to be fake news.

The AI Act will have direct effect in all the Member States of the EU. It addresses the impact of AI systems on safety, health and fundamental rights and imposes a set of dedicated requirements on the providers of high risk AI systems. In the proposal, an AI system is defined in art. 3(1) of the AIA as

“software that is developed with one or more of the techniques and approaches listed in Annex I and can, for a given set of human-defined objectives, generate outputs such as content, predictions, recommendations, or decisions influencing the environments they interact with”

The techniques and approaches listed in Annex I are:

(a) Machine learning approaches, including supervised, unsupervised, and reinforcement learning, using a wide variety of methods including deep learning;
(b) Logic- and knowledge-based approaches, including knowledge representation, inductive (logic) programming, knowledge bases, inference, and deductive engines (symbolic) reasoning and expert systems;
(c) Statistical approaches, Bayesian estimation, search, and optimization methods.

Let's first note that the definition does not concern AI as a research domain or as an attempt to imitate or improve upon human intelligence. The definition targets systems and defines them in terms of four conditions: (1) it must be a software system (so both mere computer code uploaded to github and hardware without a software component are excluded), (2) the software system must have been developed by way of one or more of the techniques in the Annex, which includes both machine learning and logic- or knowledge based systems and various other techniques that seem already implied in the machine learning approaches, (3) the system is developed based on human defined objectives, which is inevitable but important as it links to the concept of intended purpose that is key to the Act and (4) to count as an AI system for the AIA it must generate outputs that influence the environments it interacts with^31^. The latter means that an excel sheet in itself is not an AI system but might be so if it generates outputs that impact its environment, based on input from its environment. If an excel sheet is used to decide on social security benefits based on inputs about the targeted applicant, I could imagine this qualifies as an AI system. Actually this seems perfectly reasonable to me, the excel sheet is a logic based systems that performs certain operations on input data to generate an output. This output can be a recommendation and it should be clear that most RecSyss will squarely fall within the definition of AI systems in the current version of the Act.

The more interesting question is whether a RecSys is a high risk system, or rather under what conditions it could be qualified as such. This will depend on the impact they have^32^. Annex III sums up a set of contexts and applications that involve high risk, e.g., when influencing access to education, employment, social benefits or creditworthiness, and various uses in public administration, including border control and criminal justice^33^. As to marketing and advertising the AIA prohibits the use of AI systems for subliminal manipulation and exploitation of vulnerable groups if such usage “causes or is likely to cause (…) physical or psychological harm” for an individual person^34^. This may be hard to prove, but depending on how the burden of proof is distributed and on whether collective action is possible, I could imagine that certain types of RecSyss may at some point be prohibited.

If a RecSys were to be qualified as high risk, a whole range of dedicated legal obligations apply, mainly to the providers of these systems. They need to put in place a risk management system that assesses risks to health, safety and fundamental rights, both when the RS is used for its intended purpose and for reasonably foreseeable other purposes. For instance, the intended purpose of learning analytics may be to assess the progress of students, but once it is used to recommend exclusion the stakes become higher and non-discrimination could become a serious risk. The Act stipulates keen attention to the choice and curation of training data, and to various types of testing, while also emphasizing proper performance metrics, robustness, and cybersecurity, coupled with documentation and record keeping, automated logging, and post market monitoring. The provider must put in place a quality management system and ensure both by design and by way of instruction that human oversight is meaningful, effective, and practical.

In other words, the AIA restricts the kind of choices that providers of AI systems can make when leading the development of these systems, noting that the obligations often concern the backend system, the research design and the default settings. By thus skewing the choice architecture of providers, the AIA will favor resilient, robust, reliable and responsible (4R) AI systems; demanding better rather than more innovation.

## Conclusions: Deepening the Brussels Effect?

The EU legal framework will shape the development, provision and deployment of RecSyss as it shapes global economic markets. As Bradford has convincingly argued, transnational corporations wishing to compete on the internal market of the EU will adapt their behavior to stringent EU rules, because redesigning their internal processes per jurisdiction would be more costly (Bradford, 2020). It may be interesting, in that light, that corporate enterprise would prefer a level playing field that affords the development of responsible AI, noting that recently an 8.7 trillion USD investors alliance called on the EU legislature to integrate effective respect for digital rights in the DSA (Investor Alliance for Human Rights, 2022). This may also help to address naïve or malicious invocations of cost-benefit analyses (e.g., Laurer et al., 2021), which suggest that respect for human rights should be calculated and weighed against the costs of providing reliable and responsible RecSyss. This is not to suggest that compliance costs should not be assessed (see e.g., CEPS, 2022), but to situate the role of such costs; imagine if we were to compare the costs of compliance with anti-corruption legislation against the benefits of corrupt governance.

This article, however, is not about cost-benefit analyses. It is about the assumptions that must be made when developing RecSyss, more specifically about the computational, machine-consumable proxies that determine the input, learning model and the output of RecSyss. In this article, I have explained how and why the issue of proxies is key to understanding both the productive nature and the potential corruption of RecSyss, due to their behaviorist and utilitarian underpinnings. Key concepts such as “preferences” (whether latent or explicit) function as computable proxies for what individuals may desire or believe, compressing the rich and dynamic reality of human interaction to discrete sets of supposedly given (and manipulable) indicators of their future behaviors. Note that the dependencies this creates (on reductive understandings of the human condition) return in naïvely quantified cost-benefit analyses. Only if we become aware of the framing powers that are inherent in the choice and formalization of the proxies, can the far-reaching implications of such choices be called out as both foundational and political. Legislation is needed to present those who call the shots on how to choose these proxies, with a well-thought-out choice architecture that constrains developers and providers in the right direction, aiming to protect human capabilities instead of reducing human agency.

As any computer scientist knows, it is constraints that afford freedom. Without constraints, a computing system cannot “act”; computer code is all about constraints. What matters is which constraints, targeting what kind of behavior, based on what types of feedback. In this article, I have argued that constraints do not only matter for computing systems, but also for economic markets that determine what type of products and what kind of providers are successful. In turn, legal constraints are the deep code of economic markets, which are not determined by the mythological narratives of Neo-Darwinism (Sahlins, 1976) but co-constituted by the legal constraints we decide to impose on them. Legal limits are key to abolishing the provision of unreliable RecSyss that serve the preferences of advertiser intermediaries and/or the providers of very large online platforms instead of serving those in need of relevant and reliable recommendations.

## Author Contributions

The author confirms being the sole contributor of this work and has approved it for publication.

## Funding

This research was funded by the European Research Council (ERC) under the HORIZON2020 Excellence of Science program ERC-2017-ADG No 788734 and by the VUB Research Council's research mandate for my Chair on ‘Interfacing Law and Technology’.

## Conflict of Interest

The author declares that the research was conducted in the absence of any commercial or financial relationships that could be construed as a potential conflict of interest.

## Publisher's Note

All claims expressed in this article are solely those of the authors and do not necessarily represent those of their affiliated organizations, or those of the publisher, the editors and the reviewers. Any product that may be evaluated in this article, or claim that may be made by its manufacturer, is not guaranteed or endorsed by the publisher.

## Footnotes

^1^See Definition of perverse from the Cambridge Academic Content Dictionary. Cambridge University Press. Available online at: https://dictionary.cambridge.org/dictionary/english/perverse: “having the effect of being, or intended to be, the opposite of what is usually expected or considered reasonable”.

^2^Proposal for a Regulation of the European Parliament and of the Council on a Single Market For Digital Services (Digital Services Act) and amending Directive 2000/31/EC, COM/2020/825 final. Available online at: https://eur-lex.europa.eu/legal-content/en/TXT/?uri=COM%3A2020%3A825%3AFIN

^3^Regulation (EU) 2016/679 of the European Parliament and of the Council of 27 April 2016 on the protection of natural persons with regard to the processing of personal data and on the free movement of such data, and repealing Directive 95/46/EC (General Data Protection Regulation). Available online at: https://eur-lex.europa.eu/eli/reg/2016/679/oj

^4^Proposal for a Regulation of the European Parliament and of the Council laying down harmonized rules on artificial intelligence (Artificial intelligence act) and amending certain union legislative acts, COM/2021/206 final. Available online at: https://eur-lex.europa.eu/legal-content/EN/TXT/?uri=CELEX%3A52021PC0206

^5^Some versions of behaviorism avoid the reductive assumptions discussed here. Notably the work of Mead and Morris (1962) takes a different perspective, informed by modesty and ingenuity rather than arrogance and intent to manipulate.

^6^For example, framing nudging techniques as “tools that help people make better decisions “without forcing certain outcomes upon anyone” (Thaler et al., 2012, p. 428–439),” thus declaring them beneficial by definition (Jesse and Jannach, 2021).

^7^See https://adobservatory.org/

^8^See https://www.usemp.eu

^9^Proposal for a Regulation of the European Parliament and of the Council on a Single Market For Digital Services (Digital Services Act) and amending Directive 2000/31/EC, draft as amended by the Council of the EU on 18 November 2021. Available online at: https://data.consilium.europa.eu/doc/document/ST-13203-2021-INIT/en/pdf

^10^The Letter can be downloaded here: https://algorithmwatch.org/en/defend-public-interest-research-on-platforms/. Algorithm Watch requires that art. 31 should mention other sources of public interest research, notably civil society organizations and journalists. For a similar call to uphold and refine art. 31 see this Open Letter, signed by 50 organizations: https://en.panoptykon.org/fix-algorithms

^11^I would recommend (sic!) reading Harry Frankfurt's seminal *On Bullshit* to better understand how the perverse incentives of the political economy of recommender systems destroys the conditions of possibility of democratic decision making (Frankfurt, 2005).

^12^On the human labor that goes into labeling training data (see Denton et al., 2021; Jones, 2021).

^13^Social scientist Campbell had observed the same (Campbell, 1979): “The more any quantitative social indicator is used for social decision-making, the more subject it will be to corruption pressures and the more apt it will be to distort and corrupt the social processes it is intended to monitor”.

^14^The more interesting insights in the economic incentives in societies of hunters and gatherers can be found in the semial work of anthropologist Sahlins (1974), who also wrote saliently about attempts to ground human nature in naive interpretations of Darwin (Sahlins, 1976).

^15^Though I am aware of the prominence of Norman (1999) reconfiguration of Gibson's affordance theory in the context of human centric design, I am here referring to Gibson's original approach and, e.g., that of Heras-Escribano (2019), which provides for keen attention to the role of affordances in the constitution of human agency—and vice versa.

^16^Art. 4(1) GDPR: “personal data” means any information relating to an identified or identifiable natural person (“data subject”); an identifiable natural person is one who can be identified, directly or indirectly, in particular by reference to an identifier such as a name, an identification number, location data, an online identifier or to one or more factors specific to the physical, physiological, genetic, mental, economic, cultural, or social identity of that natural person.

^17^Art. 4(7) GDPR: “controller' means the natural or legal person, public authority, agency or other body which, alone or jointly with others, determines the purposes and means of the processing of personal data; where the purposes and means of such processing are determined by Union or Member State law, the controller or the specific criteria for its nomination may be provided for by Union or Member State law”.

^18^There are actually six potential legal bases available in art. 6 of the GDPR: consent, contract, vital interests of the data subject or another, a legal obligation, performance of a task in the public interest or in the exercise of public authority, legitimate interest of the controller.

^19^Art. 4(11) GDPR: “consent' of the data subject means any freely given, specific, informed, and unambiguous indication of the data subject's wishes by which he or she, by a statement or by a clear affirmative action, signifies agreement to the processing of personal data relating to him or her”.

^20^Art. 7(3) GDPR: “The data subject shall have the right to withdraw his or her consent at any time. The withdrawal of consent shall not affect the lawfulness of processing based on consent before its withdrawal. Prior to giving consent, the data subject shall be informed thereof. It shall be as easy to withdraw as to give consent”.

^21^Art. 6(1)f GDPR: “processing is necessary for the purposes of the legitimate interests pursued by the controller or by a third party, except where such interests are overridden by the interests or fundamental rights and freedoms of the data subject which require protection of personal data, in particular where the data subject is a child”.

^22^Art. 21(1) GDPR: “The data subject shall have the right to object, on grounds relating to his or her particular situation, at any time to processing of personal data concerning him or her which is based on point (e) or (f) of Article 6(1), including profiling based on those provisions. The controller shall no longer process the personal data unless the controller demonstrates compelling legitimate grounds for the processing which override the interests, rights, and freedoms of the data subject or for the establishment, exercise or defense of legal claims”.

^23^Note that consent is also required by art. 5.3 of the ePrivacy Directive, even where is concerns the processing of data that do not qualify as personal data: “Member States shall ensure that the storing of information, or the gaining of access to information already stored, in the terminal equipment of a subscriber or user is only allowed on condition that the subscriber or user concerned has given his or her consent, having been provided with clear and comprehensive information, in accordance with Directive 95/46/EC, inter alia, about the purposes of the processing. This shall not prevent any technical storage or access for the sole purpose of carrying out the transmission of a communication over an electronic communications network, or as strictly necessary in order for the provider of an information society service explicitly requested by the subscriber or user to provide the service”.

^24^Art. 12-14 impose transparency obligations on controllers, notably art. 13(1)c and 14(1)c: “the controller shall provide the data subject with all of the following information: (…) the purposes of the processing for which the personal data are intended as well as the legal basis for the processing”.

^25^Art. 5(1)b GDPR: “Personal data shall be collected for specified, explicit and legitimate purposes and not further processed in a manner that is incompatible with those purposes; (…) (“purpose limitation”)”.

^26^Art. 6(1)a GDPR: “the data subject has given consent to the processing of his or her personal data for one or more specific purposes”.

^27^Art. 7(4) GDPR: “When assessing whether consent is freely given, utmost account shall be taken of whether, inter alia, the performance of a contract, including the provision of a service, is conditional on consent to the processing of personal data that is not necessary for the performance of that contract”.

^28^Art. 22(1) GDPR: “The data subject shall have the right not to be subject to a decision based solely on automated processing, including profiling, which produces legal effects concerning him or her or similarly significantly affects him or her”.

^29^Art. 15(1)h GDPR: “The data subject shall have the right to obtain from the controller confirmation as to whether or not personal data concerning him or her are being processed, and, where that is the case, access to the personal data and the following information: the existence of automated decision-making, including profiling, referred to in Article 22(1) and (4) and, at least in those cases, meaningful information about the logic involved, as well as the significance and the envisaged consequences of such processing for the data subject”.

^30^Report from the Commission to the European Parliament, the Council and the European Economic and Social Committee. Report on the safety and liability implications of Artificial Intelligence, the Internet of Things and robotics, Brussels, 19.2.2020 COM(2020) 64 final, Available online at: https://eur-lex.europa.eu/legal-contenten/TXT/?qid=1593079180383&uri=CELEX%3A52020DC0064

^31^EU legislation is co-authored by the European Commission (EC), the Council of the EU (representing the member states) and the European Parliament. The draft I discuss has been written by the EC. The Council has proposed various amendments, one of which concerns the definition. Key changes are: (1) the notion of “software” is removed, the definition now simply speaks of a “system,” (2) an additional condition has been added, namely that the system should achieve its objectives by way of “learning, reasoning, or modeling.” The first seems a wise choice as it makes the Act more future proof; the second may seriously restrict the scope of the Act, excluding what the Council qualifies as “more classic software systems and programming” (amendment of recital 6) (see Bertuzzi, 2021).

^32^For the definition of high risk systems, see art. 6 AI Act which refers to Annex II (mainly focusing on safety and health risks) and Annex III (mainly focusing on risks to fundamental rights).

^33^Title III, chapter 2, art. 8–15 AI Act.

^34^Art. 4 AI Act.
